# Retroperitoneal angioleiomyomatosis

**DOI:** 10.1007/s13224-020-01404-7

**Published:** 2020-12-23

**Authors:** Luz Angela Torres-de la Roche, Rajesh Devassy, Ghaith Makhlouf, Johannes San Juan, Jennifer Eidswick, Rudy Leon De Wilde

**Affiliations:** 1 University Hospital for Gynecology, Pius Hospital, Carl von Ossietzky University, Oldenburg, Germany; 2 Department of Obstetrics and Gynecology, Dubai London Clinic and Speciality Hospital, Jumeirah St - Umm Suqeim-2, Dubai, United Arab Emirates

**Keywords:** Intravenous leiomyomatosis, Laparoscopy, Minimally invasive surgery, Angiomyoma, Immunomarkers

## Abstract

Retroperitoneal intravenous leiomyomatosis is a rare benign tumor that can spread through veins carrying significant morbidity. The challenge of its management lies within the complexity of completely excising the tumor, which if carried out improperly can result in neurological or vascular complications requiring complex reparative surgeries. Here we present the successful resection of a retroperitoneal angio-leiomyoma by combining laparoscopic route, micro-surgical techniques and modern endoscopic tools.

**Electronic supplementary material:**

The online version of this article (10.1007/s13224-020-01404-7) contains supplementary material, which is available to authorized users.

## Introduction

Intravenous leiomyomata (IVL) are rare benign vascular neoplasms characterized by intraluminal growth of smooth muscle into either venous or lymphatic vessels outside the limits of myoma. Uterine IVL can extend through the veins, with inferior vena cava extension or pulmonary and heart metastasis, carrying significant morbidity [1–3]. Retroperitoneal development is another unusual growth pattern of uterine leiomyomas and especially of IVL. Retroperitoneal neural soft tissue tumors or angio-sarcoma lesions should be considered as differential diagnosis [2]. With regard to their pathologic origin, it is unclear whether these retroperitoneal lesions represent metastatic or synchronous primary lesions and whether they arise from hormonally sensitive smooth muscle elements. In immunohistochemical studies, IVL tumors have been reported to express different cell markers (p-16, or Cyclin 1 and present CD34 and D2-40 and cytoplasmic phosphorylated-Rb CD31,) and smooth muscle markers (Desmin and SMA). Recent molecular cytogenetic studies describe a monoclonal proliferation with genetic alterations usually seen in mesenchymal tumors, mainly 1p, 22q, 2q, 1q,13q and 14q aberrations, supporting the theory that postulates IVL arise from intravascular projections of uterine myoma [1, 4]. Most of the patients with an IVL present in the 5th and 6th decades of life with symptoms related to tumor localization. Surgical resection is often curative, but recurrences have occasionally been reported [3, 4]. The challenge of IVL lies within the complexity of diagnosis and completely excising the tumor, which if carried out improperly may result in neurological or vascular complications requiring complex reparative surgeries.

## Case presentation

**Fig. 1:**
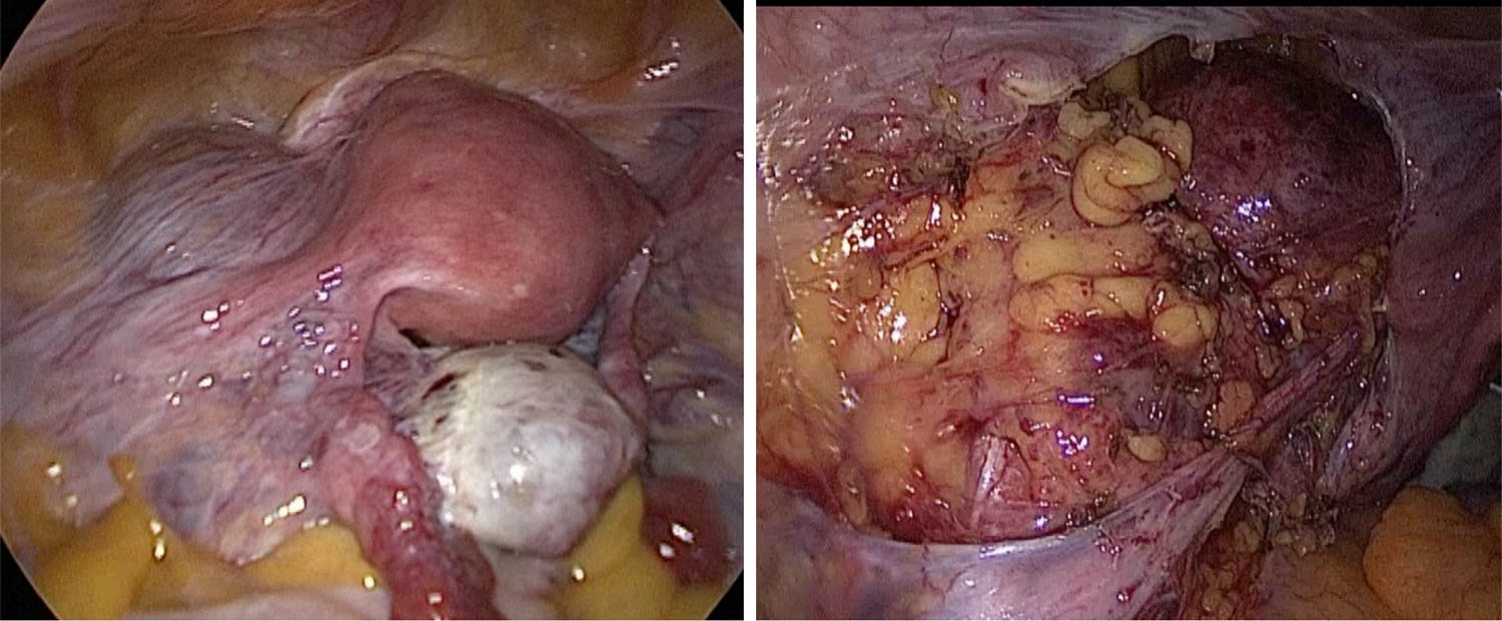
Outline of the retroperitoneal mass upon laparoscopy

**Fig. 2:**
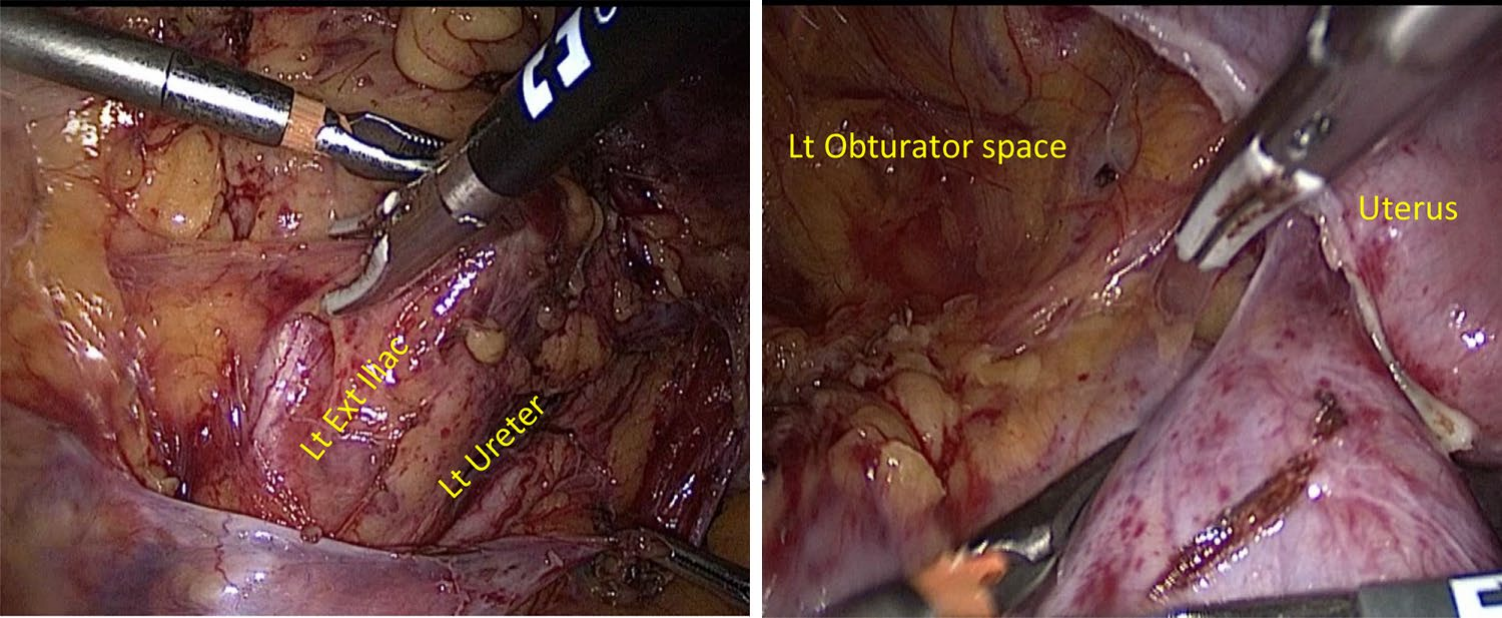
Releasing superior-lateral border of the tumor from the left external Iliac artery and Obturator with Ligasure™

**Fig. 3:**
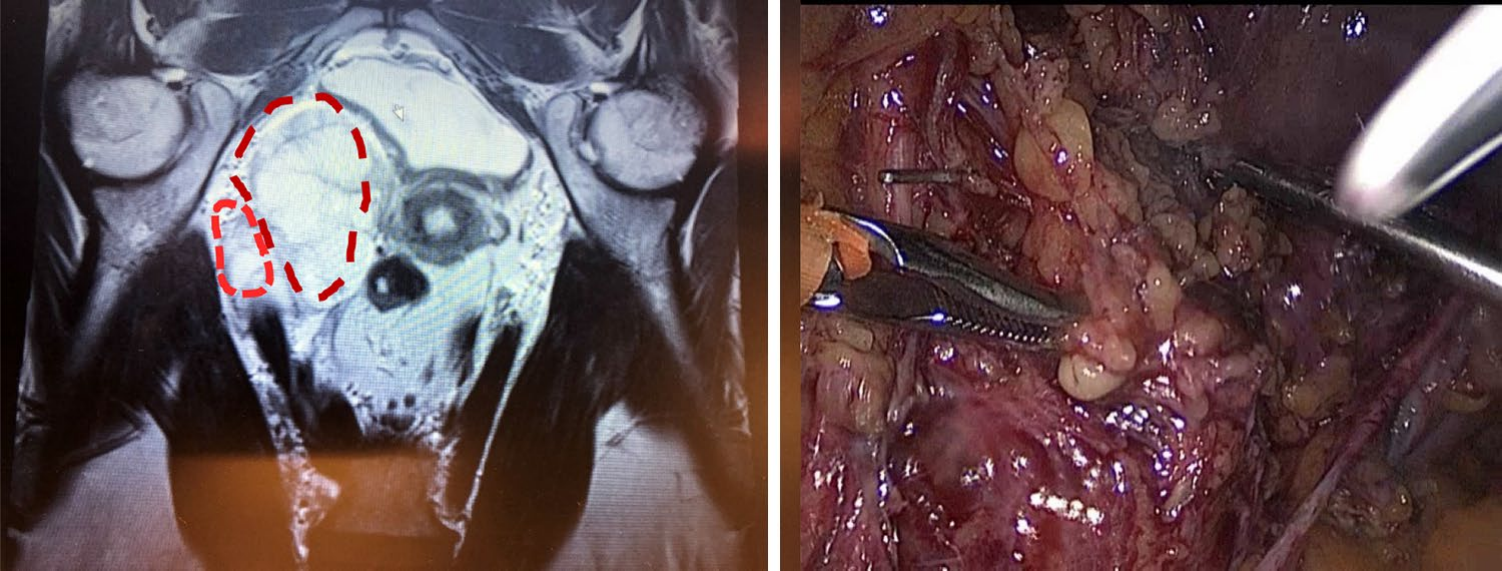
Secluding the cephalic vascular attachments anterior to the external Iliac vessels possible branches of anterior division of IIA and/or tributaries of Iliolumbar artery

**Fig. 4:**
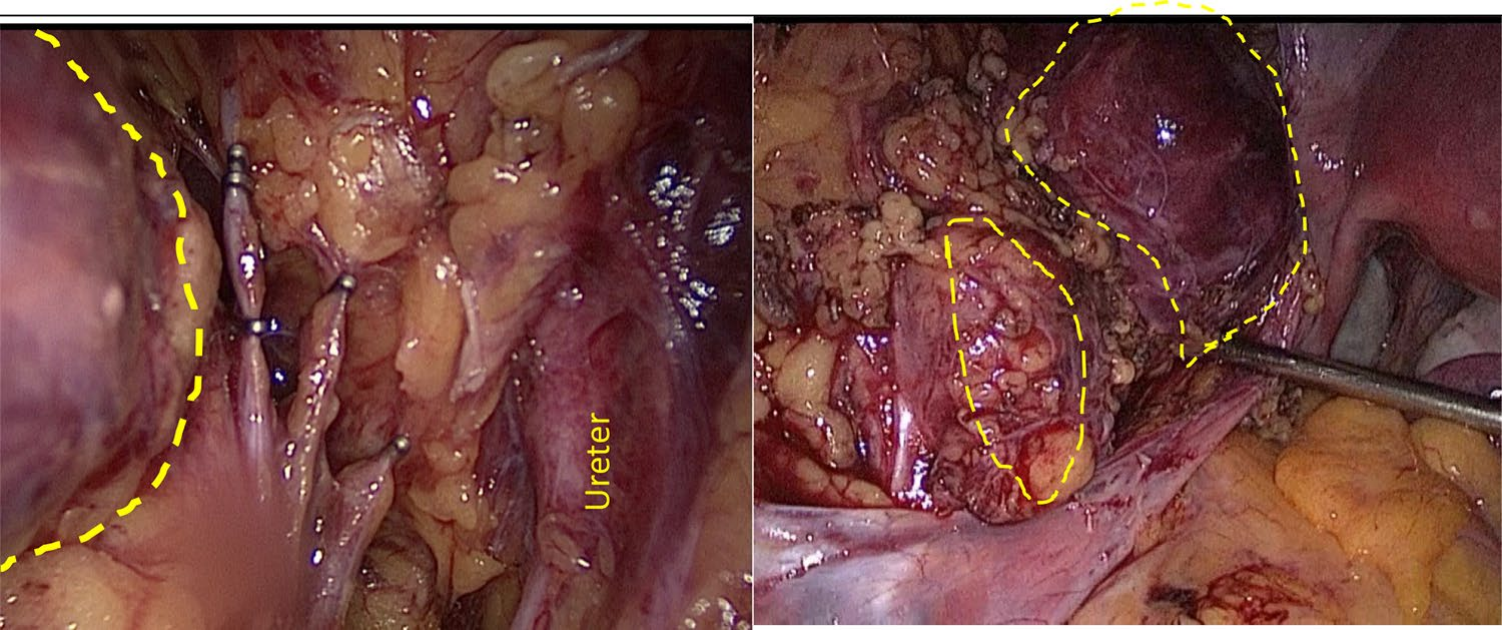
Isolation and vascular clipping with Endo Clip TM of the branches supplying the tumor from uterine artery, medially isolated ureter is well appreciated

**Fig. 5:**
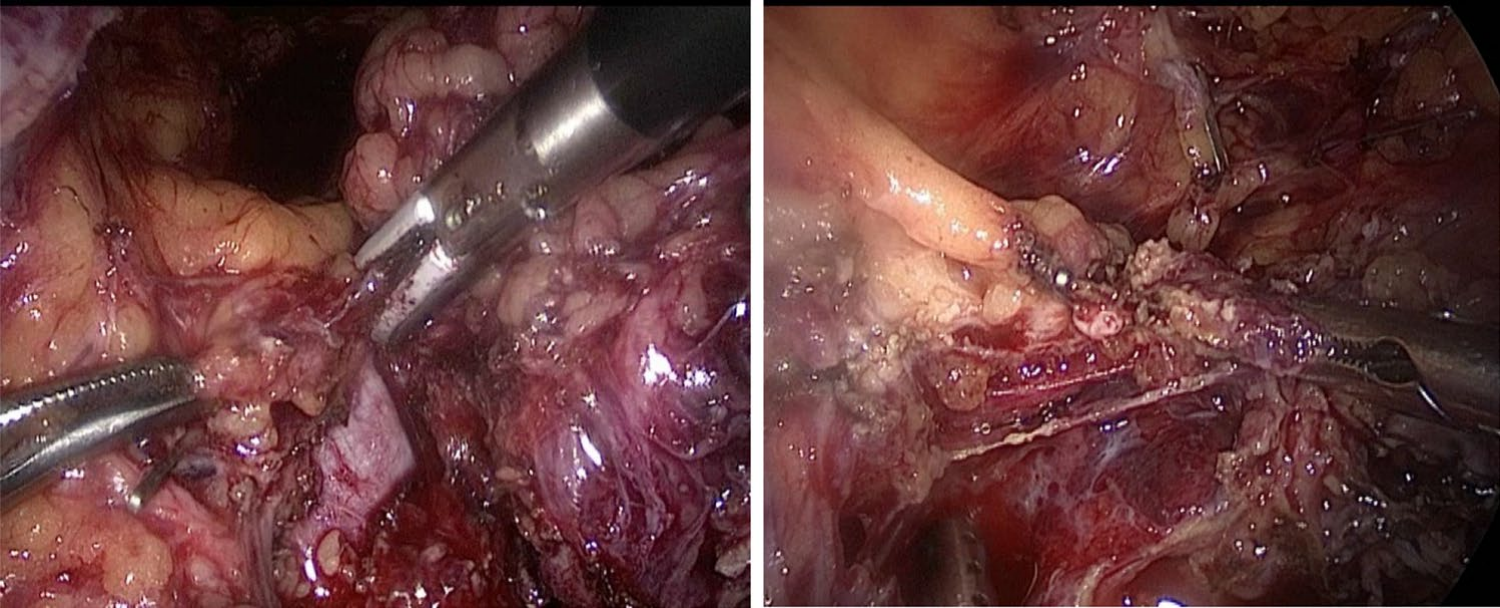
Medial attachments of vascular branches arising out of the external iliac divided with Ligasure ™ in between the vascular Endo Clip ™

**Fig. 6:**
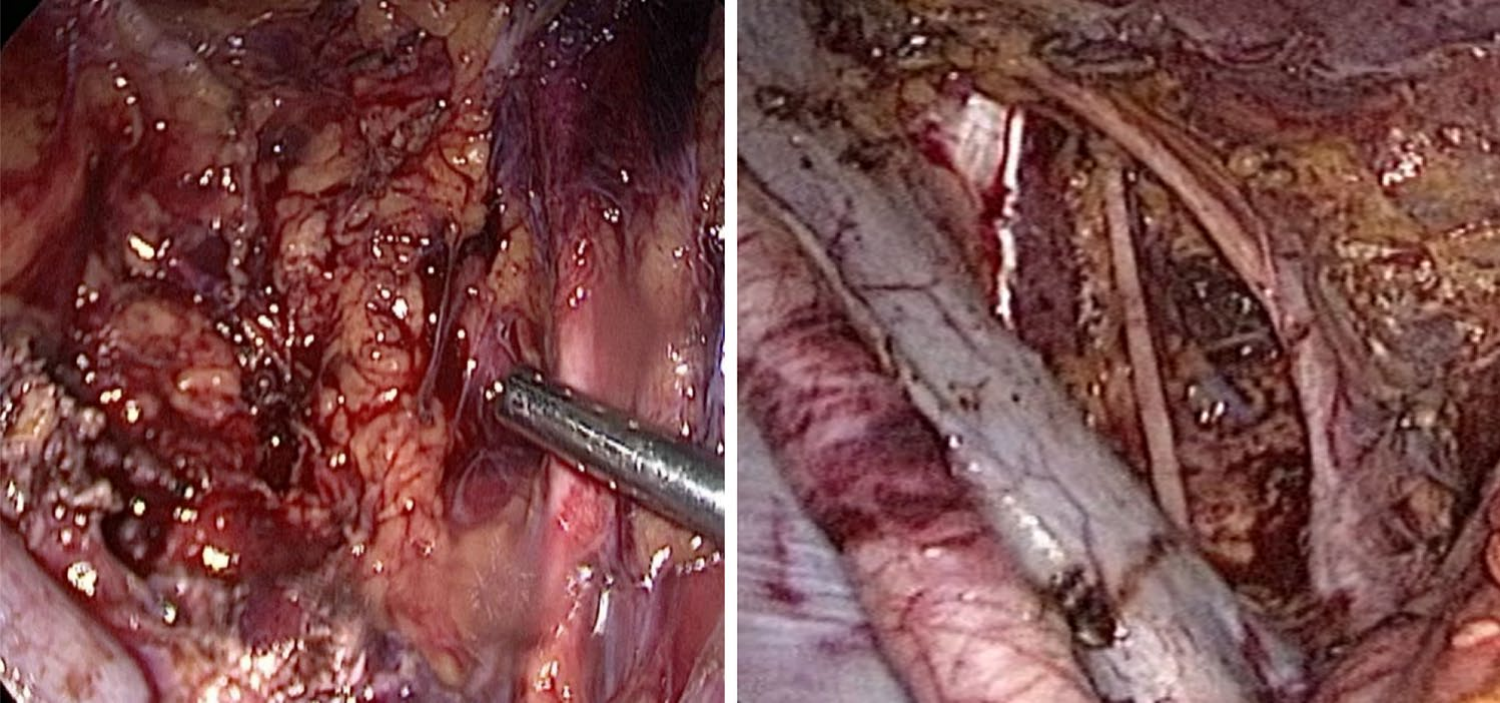
Final hemostasis achieved confirmed in submersed LRS with hemostatic and adhesion barrier Veriset™ matrix patch

A 35 year-old patient with two children underwent laparoscopic retroperitoneal pelvic tumor excision at the Dubai London Clinic and Speciality Hospital. The patient presented with chronic left leg pain for many years. At admission, ultrasound examination and MRT image findings were described as a mucinous cystadenoma of the left ovary (9.2 × 5.6 cm with relative irregular outline and filling the pouch of Douglas), with the differential diagnosis of a broad ligament leiomyoma with intrinsic tumor degeneration and necrotic features. By combining laparoscopic route, in-bag morcellation and micro-surgical technics, it was possible to ensure complete resection of the retroperitoneal tumor without intraoperative vascular or neural complications (Figs. 1, 2, 3, 4, 5 and 6; Ligasure ™, Sonicision ™, Endo Clip ™, Endo Catch ™, Veriset ™ are Registered trade marks of Medtronic). At histological examination, the cytology of peritoneal fluid showed mild subacute inflammation with activated mesothelial cells, suggestive of exudative peritoneal effusion. Sections of the soft tissue neoplasm consisted of cellular sheets and irregular fascicles spindled cells with formation of glomeruloid architecture and epithelioid features. The lesion demonstrated numerous anastomosing structures resembling blood vessels covered by endothelium-like cells embedded in fibrous stroma. No evidence of malignancy or tumor necrosis was reported. The tumor was positive for immunomarkers CD31, CD34, D2-40, CD68, Ki-67 (1–2% positive nuclear staining) as well for smooth muscle markers SMA (Smooth muscle actin) and Desmine. HHV-8, S100, Synaptophysin and Calretinin were negative. The postoperative course was uneventful.

## Discussion

Multiple leiomyomatous masses are usually seen in the pelvic retroperitoneum in postmenopausal women with a concurrent uterine leiomyoma or a history of uterine leiomyoma. Rarely, the tumor exhibits smooth muscle proliferation within the vascular and lymphatic spaces of the myometrium, known as IVL, which in 30-80% of cases propagates through its adjacent venous structures beyond the pelvis [3, 4]. Affected patients may be asymptomatic or, if the tumor is limited to the pelvis, they may present with vaginal bleeding, pelvic or abdominal pain. In metastasizing disease, fatigue, ascites, peripheral edema, dyspnea, syncope, congestive heart failure and deep vein thrombosis may be present. When IVL presents as a retroperitoneal mass in the proximity of nerves, half of patients seek medical help due to chronic pelvic pain [1–4]. In contrast, we present a case of IVL diagnosed in a young patient (35 years-old) who, furthermore, presented with chronic leg pain not usually described in the literature.

IVL are not usually diagnosed before surgery. At simple ultrasound imaging, IVL are not easy to distinguish; at color-duplex examination, IVL presents as a heterogeneous vascular mass with high resistance. On axial contrast-enhanced CT-images, lesions resemble a ‘sieve’ and, on 3D volume rendered CT-images, a ‘luffa sponge’. MRI imaging tumors can be seen as hypertensive and isointensive to skeletal muscle [5]. In this case, it was not possible to suspect the vascular anomaly of the tumor because no vascular Doppler was performed and at MRI the tumor was described as an ovarian mass (mucinous cystadenoma). Therefore, the diagnosis was made postoperatively by means of histopathology and cell markers examination. As reported in previous studies [1], the tumor was positive for immunomarkers CD31, CD34, D2-40, CD68, Ki-67 (1–2% positive nuclear staining) as well for smooth muscle markers SMA and Desmine.

Surgery is the treatment of choice and is critical to assuring complete IVL resection to reduce recurrences. Percutaneous embolization could be used to reduce the size and vascularization of the tumor prior to surgical excision [3, 4]. Despite the excellent prognosis, long-term imaging follow-up of patients is recommended. If any tumor is left in pelvic or affected veins, a bilateral oophorectomy should be considered for premenopausal women, since estrogen stimulates tumor growth [2, 4]. In our case, a minimally invasive approach, by combining laparoscopic route, in-bag morcellation and micro-surgical technics, facilitated the complete tumor resection without complications, opening a feasible and safe avenue for the management of this type of tumor.

## Conclusion

Retroperitoneal angio-leiomyoma is an extremely rare and yet a benign tumor. The complexity of its surgical treatment lies in its localization nearby to vascular and nerve structures and the high-risk of perioperative complications. However, successful resection of a giant retroperitoneal angio-leiomyoma was achieved by combining laparoscopic route, micro-surgical techniques and modern endoscopic tools, including vascular clips, ultrasonic dissectors and in-bag morcellation. We encourage surgeons to offer this approach to their future patients.

## Electronic supplementary material

Below is the link to the electronic supplementary material.

Supplementary material 1 (MP4 180284 kb)
